# Hydroxycamptothecin induces apoptosis and inhibits tumor growth in colon cancer by the downregulation of survivin and XIAP expression

**DOI:** 10.1186/1477-7819-11-120

**Published:** 2013-05-30

**Authors:** Bojian Fei, Alfred L Chi, Yuan Weng

**Affiliations:** 1 Department of Surgical Oncology, No.4 people’s hospital, Wuxi City, 214062, PR China; 2 Department of Thoracic and Cardiovascular Surgery, No.4 people’s Hospital, Wuxi City, 214062, PR China; 3 CHI Scientific, Inc., 63 Great Road, Maynard, MA, 01754, USA

**Keywords:** 10-Hydroxycamptothecin, 5-fluorouracil, Colon cancer, Chemotherapy

## Abstract

**Background:**

10-Hydroxycamptothecin (10-HCPT), isolated from a Chinese tree *Camptotheca acuminate*, inhibits the activity of topoisomerase I and has a broad spectrum of anticancer activity *in vitro* and *in vivo*. It has been shown that HCPT is more active and less toxic than conventional camptothecins and can induce cancer cell apoptosis. However, the mechanisms of HCPT-induced apoptosis in colon cancer cells remain unclear. In this study, we investigated the effects of HCPT on apoptosis of colon cancer and underlying mechanism.

**Methods:**

Cell proliferation was measured by MTT (3-[4,5-dimethylthiazol-2-yl]-2,5-diphenyl tetrazolium bromide) assay, and apoptosis was measured using terminal deoxynucleotidyl transferase-mediated dUTP-biotin nick end labeling (TUNEL) assay. Expression of genes was detected using real-time reverse transcription-polymerase chain reaction (real time-PCR) and Western blot. Tumor growth *in vivo* was evaluated using a nude mouse xenograft model.

**Results:**

HCPT could significantly inhibit cell proliferation and induce apoptosis in colon cancer SW1116 and Colo 205 cells in dose- and time-dependent manners. HCPT treatment activated the activities of caspase 3, 7, 8 and 9, downregulated the expression of survivin, survivinΔEx3, survivin-3B and XIAP, and upregulated expression of surviving 2B. Moreover, the combination of HCPT and 5-fluorouracial (5-FU) synergistically induced apoptosis and downregulated the expression of survivin and XIAP. Knockdown of survivin and XIAP by siRNA sensitized colon cancer to HCTP-induced apoptosis. Furthermore, HCPT treatment significantly inhibited SW1116 xenograft tumor growth.

**Conclusions:**

Our results elucidate new mechanisms of HCPT antitumor by the downregulation of survivin and XIAP expression. The combination of HCPT with 5-FU or IAP inhibitors may be a potential strategy for colon cancer treatment.

## Background

Colorectal cancer (CRC) is one of the most common cancers in the world [1,2]. Even with the development of biological agents and chemotherapy, colorectal cancer (CRC) is still a major cause of mortality in cancer patients. Therefore, developing new therapeutic drugs for advanced colon cancer remains a challenge. DNA topoisomerases (Topo) are enzymes that regulate the overwinding or underwinding of DNA and are essential to maintaining the helical structure of DNA [3]. Topo-I cuts a single strand of DNA to allow the relaxation of torsional stresses before re-annealing. Studies have shown that Topo-I is highly expressed in around half of colorectal cancers [4] and that genomic amplification of Topo-I is correlated with the increased RNA and protein expression of Topo-I in colorectal cancer [5]. Therefore, Topo-I has become a target for cancer therapy [6,7].

A natural indole alkaloid extracted from a Chinese tree *Camptotheca cuminata*, 10-hydroxycamptothecin (HCPT) is a topoisomerase I-specific inhibitor [3,6-8]. The formation of a cleavable drug, Topo-I-DNA complex, results in the lethal breakage of double-strand DNA and cell death [3,6-8]. HCPT has a broad spectrum of anti-tumor activity against a panel of solid tumors in the *in vitro* and *in vivo* animal models and human cancer patients [8-10]. However, *de novo* or acquired clinical resistance to camptothecins (CPTs) is common [11-14]. Little is known about the mechanisms of HCPT anti-tumor effects and the development of drug resistance in human colon cancer.

HCPT exhibits strong anti-cancer effects and is less toxic than CPT [12]. Previous studies indicate that HCPT and its analogs can stabilize the reversible covalent DNA-Topo-I complex, resulting in apoptosis of cancer cells [10,11]. HCPT exhibits high S phase-specific cytotoxicity and induces G2-M cell cycle arrest [3,15]. Further studies show that HCPT-induced replication fork collision contributes to S phase cytotoxicity. HCPT exhibits an inhibitory effect on the phosphorylation of histone H1 and H3 in murine hepatoma cells, which results in its specific cell killing effect [16]. It also exhibits a differentiation inducing effect in human HepG2 cells [17]. Studies show that camptothecin inhibits gastric cancer growth and induces apoptosis by the upregulation of p53, p21Waf1/Cip1 and p27Kip1 and the downregulation of Bcl-2 and Bcl-XL [18]. These studies have suggested that the anti-cancer function of HCPT is not consistent with the inhibition of Topo-I activity, which implies that additional mechanisms are involved in HCPT-induced cell death.

Accumulating data show that HCPT can induce apoptosis in multiple cancers [19] and can inhibit metastatic colorectal cancer [20,21]. The studies have shown that the combination of 5-fluorouracil (5-FU) with Topo-I inhibitor remains as one of the main treatments for advanced cancer [21,22]. However, the mechanisms of the combination of HCPT and 5-FU remain largely unknown. In this study, we investigated the effects of HCPT alone or in combination with 5-FU on colon cancer growth and the underlying mechanisms involved.

## Methods

### Cell culture and reagents

The human colon cancer cell lines SW1116 and Colo 205 were obtained from ATCC (Rockville, MD, USA) and maintained in a Roswell Park Memorial Institute (RPMI)-1640 medium containing 10% fetal bovine serum (FBS), 100 U/ml penicillin and 100 μg/mL streptomycin (Gibco BRL, Life Technologies, NY, USA). HCPT and 5-FU purchased from Sigma (St. Louis, MO, USA) were dissolved in dimethyl sulfoxide (DMSO) and stored at 4°C.

### Cell proliferation assay

Cell proliferation was determined using 3(4,5 dimethylthiazol)-2,5 diphenyltetra-zolium (MTT) assay; 100 μL SW1116 and Colo 205 cells in exponential growth at 1 × 10^4^/mL were seeded into flat-bottomed 96-well plates (NUNC) 24 hours prior to the drug treatment. Cells were treated with 0.1 μg/mL to 10 μg/mL HCPT in triplicate for 48 hours. After washing, the medium was replaced by 100 μL RPMI 1640 (GIBCO) medium containing 1 mg/mL MTT (Sigma). After 4 hours, the plates were centrifuged at 800 × g for 5 minutes, the MTT medium was removed, and the purple formazan crystals were dissolved in 200 μL of warm DMSO per well. After 10 minutes, the plates were read on the microplate reader (American Bio-Tek) at 570 nm. The cells without drugs were used as the control. The survival of the cells was expressed as the percentage of untreated control wells. Assays were performed on three independent experiments.

### Transfections of survivin shRNA and X-linked inhibitor of apoptosis protein shRNA

Colon cancer SW1116 cells (2 × 10^5^ per well) were seeded on a six-well tissue culture plate for 24 hours prior to transfection. The SW1116 cells were transfected with 50 pmols survivin siRNA (Santa Cruz Biotechnology, Santa Cruz, CA, USA) or X-linked inhibitor of apoptosis protein (XIAP) siRNA (Santa Cruz Biotechnology) using siRNA Transfection Reagent (Santa Cruz Biotechnology, sc-29528) for 6 hours according to the manufacturer’s instruction. Then cells were incubated with HCPT at 1 mL of normal growth medium for an additional 24 or 48 hours. Cells were harvested for apoptosis analysis using TUNEL or for western blot analysis.

### Apoptosis assays

Apoptosis was assessed by 2´-deoxyuridine, 5´-triphosphate (dUTP) labeling of DNA nicks with terminal deoxynucleotidyl transferase (TUNEL). Colon cancer cells (3 × 10^5^/well) were inoculated into 6-well plates with previously placed glass slides. After 24 hours, cells were treated with HCPT in the presence or absence of capase-3 inhibitor z-DEVD-fmk. At 24 hours and 48 hours after the treatment with HCPT, glass slides with cancer cell growth were fixed with 4% polyformaldehyde. The TUNEL assay was performed according to the instructions in the Apoptosis Detection Kit (Boehringer-Mannheim, Germany). Briefly, after washing twice with pH 7.4 PBS, 50 μL TUNEL reaction solutions were added to the well, and then incubated at 37°C for 2 hours. After the substrate had reacted, stained cells were examined under the light microscopy. Apoptotic cells were scored and expressed as the number of positively stained cells per 400 cells (n = 5).

### Real-time PCR

After drug treatment, cells were harvested for RNA extraction. RNA was prepared using the RNeasy mini kit along with RNase-free DNase set to get rid of any traces of DNA contamination (QIAGEN, Valencia, CA, USA). cDNA was prepared from 500 ng of RNA (TaqMan Reverse Transcription Reagents, ABI/Roche, Branchburg, NJ, USA), based on the manufacturer’s instructions. The template cDNA (12.5 ng/sample) for control and HCPT was mixed with Syber green master mix (Life Technologies, Grand Island, NY, USA). Mixtures were aliquoted into 96-well optical reaction plates (ABI) along with the forward and reverse primers for each target gene. Samples were run in triplicates. Primers were designed using ABI primer express software. Survivin forward primers: 5′-AGAACTGGCCCTTCTTGG AGG-3′; reverse: 5′-CTTTTTATGTTCCTCTATGGGGTC-3′; XIAP forward primers: 5-AA TAGTGCCACGCAGTCTACA-3′, reverse: 5′-CAGATGGCCTGTCTAAGGCAA-3′; GAPDH forward primers: 5′-TG TGGGCATCAATGGATTTGG-3′, reverse: 5′-ACACCA TGTATTCCGGGTCAAT-3′. Survivin-ΔEX3 forward primers: 5 ′-ATGACGACCCCATG CAAAG-3′ and reverse: 5-GGTGGCACCAGGGAATAAAC-3′; survivin-2B forward primers: 5′-GCGGATCACGAG AGAGGA-3′ ‘and reverse: 5′-TCTCCGCAGTTTCCTCA AAT-3′; Survivin-3B forward primers: 5′-CCTTTCTGTCAAGAAGCAGTTTG-3′, reverse: 5′-TGCTAACAGAGCTCTCTCAATTT-3′; Survivin-2α forward primers:5′-CTACATTC AAGAACTGGCCCTT-3′; reverse, 5′-CAGCTCCTTGAAGCAGAAGAA-3′. Data were analyzed using the comparative cycle threshold (*C*_T_) method, by which the fold change of target gene samples (HCPT treatment) that was normalized to the housekeeping gene (beta-actin) relative to the calibrator (control) is calculated using the 2^-ΔΔCT^ equation, where ΔΔ*C*_T_ = Δ*C*_T_ (sample) - Δ*C*_T_ (calibrator), and Δ*C*_T_ is the *C*_T_ value of the target gene subtracted from the *C*_T_ value of the housekeeping gene. All the values were determined in the exponential phase of the reactions.

### Western blot analysis

Colon cancer cells were lysed with the lysis buffer (50 mM Tris–HCl (pH 7.5), 250 mM NaCl, 0.1% NP40, 5 mM EGTA containing 50 mM sodium fluoride, 60 mM β-glycerol-phosphate, 0.5 mM sodium vanadate, 0.1 mM phenylmethylsulfonyl fluoride, 10 μg/mL aprotinin, and 10 μg/mL leupeptin). Protein samples were electrophoresed in a 10% denaturing SDS gel and transferred to Immobilon-P membrane (Millipore, Bedford, MA, USA). The blots were first incubated with specific primary antibodies XIAP, survivin, c-IAP-1, c-IAP-2, cleaved Caspase-3, 8, 9, cytochrome c antibodies (Cell Signaling Technology, Danvers, MA, USA), then reacted with a peroxidase-conjugated secondary antibody (Santa Cruz Biotechnology), and finally visualized by enhanced chemiluminescence (Amersham, Piscataway, NJ, USA).

**Figure 1:**
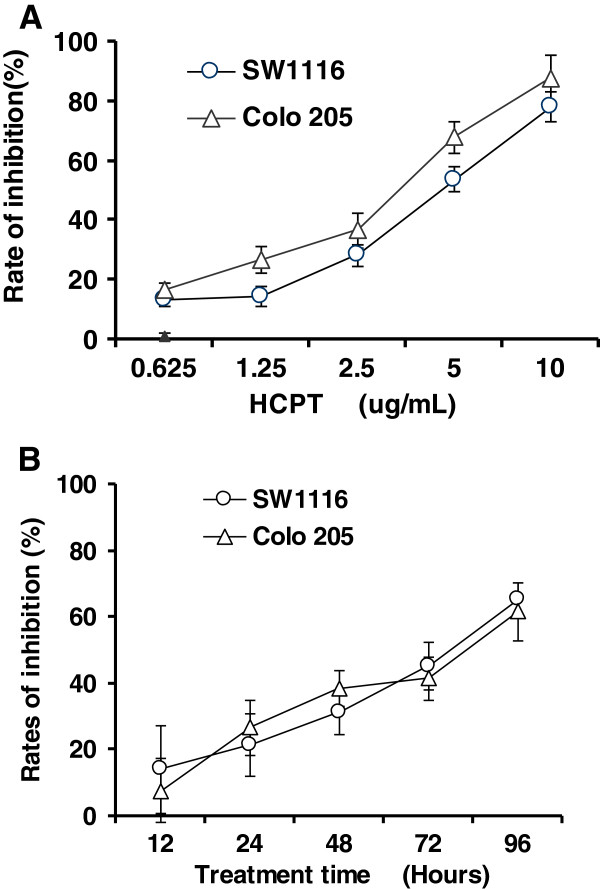
**HCPT inhibits cell proliferation.** Colon cancer cells were treated with 10-hydroxycamptothecin (HPCT) at the indicated dose (**A**) or at the indicated time (**B**). Cell inhibition was measured by MTT assay. The data are expressed as means ± SD from three independent experiments.

### Colon tumor xenograft models and treatment

Four-week-old female BALB/nude mice from Animal Experimental Center of Shanghai Institute of Biological Sciences (Shanghai, China) were maintained in a sterilized animal room in our animal facility. Colon cancer cells SW1116 (1 × 10^6^) were injected subcutaneously (s.c.) into the flank of BALB/nude mice. The mice either received a daily intrapertioneal (i.p.) injection of HCPT (7.5 mg/kg body weight) or a 5-FU (10 mg/kg body weight) separately or in combination after tumors reached a size of 50 mm^3^ to 60 mm^3^. Tumor sizes were measured by a caliper weekly. Tumor tissues were harvested at the end of the experiments. Animal protocols were approved by the Animal Care and Facilities Committee (ACFC) of No.4 People’s Hospital of Wuxi City, China.

### Statistical analysis

Unless indicated otherwise, all experiments were conducted at least thrice with at least five mice per group. Results, expressed as means ± SD, were analyzed for differences between the two groups using a two-tailed *t*-test with assumption of equal variances, with a *P-*value of <0.05 deemed significant. One-way analysis of variance (ANOVA) was used to compare the results of three or more groups.

## Results

### HCPT inhibits colon cancer proliferation

The effect of HCPT on the viability of colon cancer cells was determined by the MTT assay. HCPT exhibited strong cytotoxic effects on colon cancer cells. HCPT inhibited cell proliferation of colon cancer in time- and dose-dependent manners (Figure 1A and 1B). HCPT had similar inhibition effects on SW1116 and Colo 205 cells. The IC50 of HCPT on SW1116 and Colo 205 cells were 3.3 μg/mL and 3.8 μg/mL respectively. These results suggest that HCPT could inhibit cell proliferation.

### HCPT induces apoptosis in colon cancer cells

**Figure 2:**
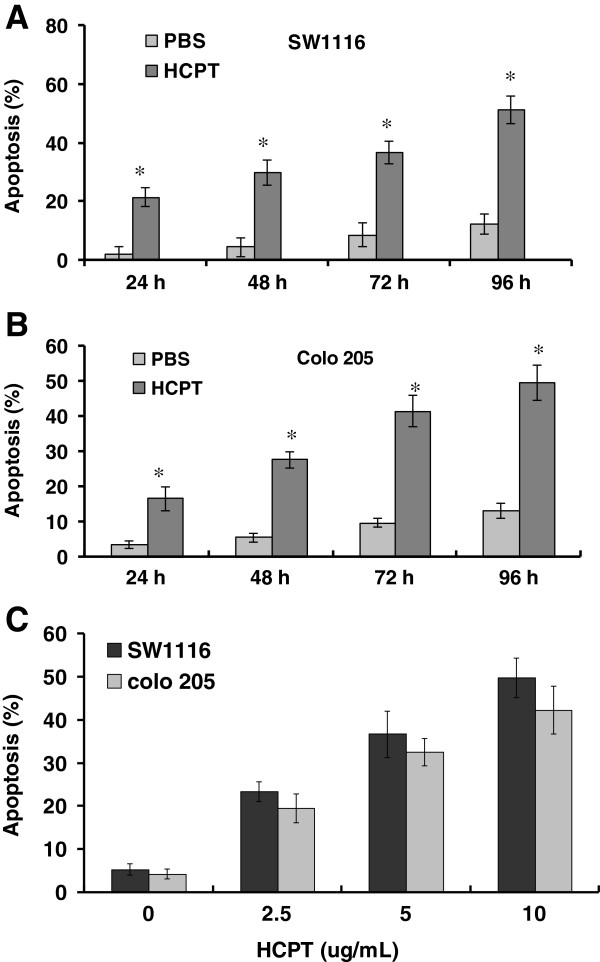
**HCPT induces apoptosis in colon cancer cells.** SW1116 **(A)** and Colo 205 (**B**) were treated with 10-hydroxycamptothecin (HCPT) (2.5 μg/mL) at the indicated time. Apoptosis was determined by terminal deoxynucleotidyl transferase-mediated dUTP-biotin nick end labeling (TUNEL) staining. The data represent the means ± SD from three independent experiments; ^*^P <0.01, compared to the PBS group. **(C)** SW1116 and Colo 205 were treated with HCPT (2.5 μg/mL) at the indicated dose for 48 hours. Apoptosis was determined by TUNEL staining. The data represent the means ± SD from three independent experiments; ^*^P <0.01, compared to PBS treatment.

Six hours after exposure to HCPT, colon cancer cells SW1116 and Colo 205, began to show morphologic features of apoptosis. The apoptotic cells increased following a prolongation of exposure time to drugs. The features of apoptosis that were observed in SW1116 cells, included cell shrinkage, cytoplasmic blebs, condensation of chromatin, nuclear condensation, fragmentation of the nucleus and the formation of apoptotic bodies (data not shown). TUNEL staining showed that HCPT induced apoptosis in SW1116 and Colo 205 cells in a time- and dose-dependent manner (Figure 2A-2C). After 48 hours of exposure to 5 μg/mL HCPT, the apoptotic rates of SW1116 and Colo 205 were 36.4% and 32.7% respectively. After the treatment of colon cancer cells with 2.5, 5 and 10 μg/mL HCPT, the apoptotic rates of SW1116 were 23.4%, 36.7% and 49.8% respectively.

### HCPT activates intrinsic and extrinsic apoptotic pathways

**Figure 3:**
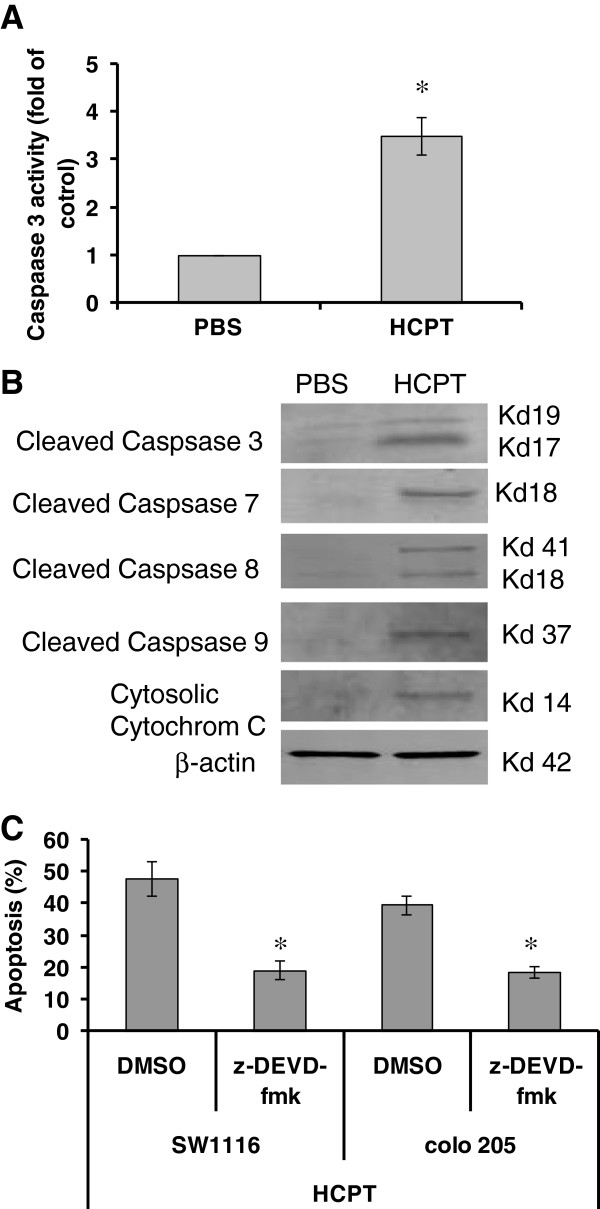
**HCPT activates the intrinsic and extrinsic apoptotic pathways. (A)** 10-hydroxycamptothecin (HCPT) increased the activity of caspase 3. SW1116 cells were treated with 2.5 μg/mL HCPT for 48 hours. Cells were harvested for caspase 3 activity assay. The data are the means ± SD from two independent experiments. ^*^P <0.01, compared to PBS group. (**B**) HCPT activated the intrinsic and extrinsic apoptotic pathways. SW1116 cells were treated with 2.5 μg/mL HCPT for 48 hours. Protein expressions were determined by western blot. (**C**) HCPT induces apoptosis in a caspase-dependent manner. SW1116 cells were pre-treated with z-DEVD-fmk for 2 hours and then treated with HCPT (10 μg/mL) for another 48 hours. Apoptosis was determined by terminal deoxynucleotidyl transferase-mediated dUTP-biotin nick end labeling (TUNEL) staining. The data represent the means ± SD from three independent experiments. ^*^P <0.01, compared to DMSO groups.

To characterize the signaling pathways by which HCPT induces apoptosis in colon cancer cells, we further determined the activities of caspase-3 in the HCPT-treated SW1116 cells. The data revealed that HCPT significantly increased the activity of caspase 3 in SW1116 cells (Figure 3A). To further investigate if HCPT activated intrinsic and extrinsic pathways during apoptosis, we determined the expression of the cleavage of caspase-3, caspase-7, caspase-9 (a caspase in the intrinsic pathway), caspase-8 (a caspase in the extrinsic pathway) and poly (ADP-ribose) polymerase (PARP). Western blot showed that HCTP treatment increased the expression of cleaved caspases and the release of cytochrome c (Figure 3B). To determine whether HCPT induce apoptosis through a caspase-dependent manner, we pre-treated SW1116 cells with caspase 3 inhibitor z-DEVD-fmk for 2 hours and then treated them with HCPT for another 48 hours. We

 found that z-DEVD-fmk treatment significantly inhibited HCPT-induced apoptosis of colon cancer cells (Figure 3C). The data indicate that HCPT induces apoptosis of colon cancer cells through both extrinsic and intrinsic apoptotic pathways.

### HCPT inhibits expression of XIAP and survivin in colon cancer cells

Inhibitors of apoptosis (IAPs) are endogenous inhibitors of caspases and are related to chemo-resistance in some cancer cells, including colon cancer [2-6]. Furthermore, the targeted downregulation of XIAP or survivin genes has been shown to directly sensitize cancer cells to apoptosis induced by various conventional chemotherapeutic drugs

**Figure 4:**
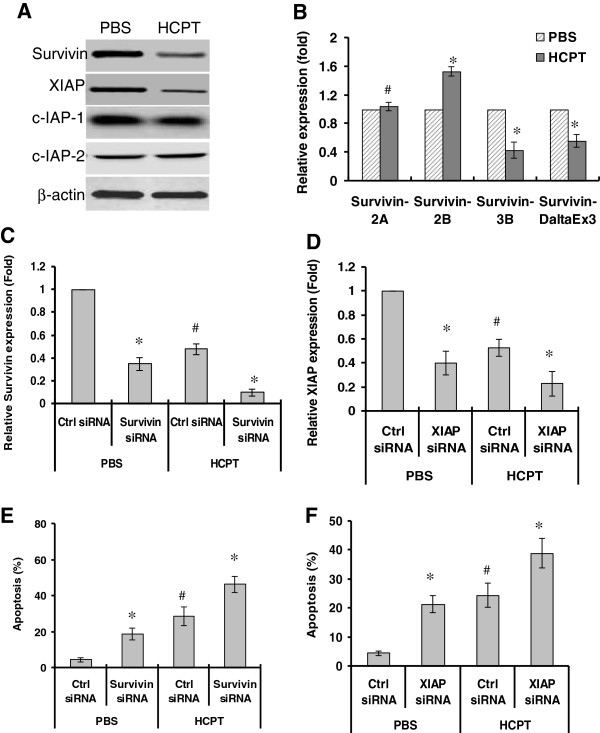
**HCPT downregulates the expression of survivin and XIAP and in colon cancer cells.** (**A**) SW1116 cells were treated with 10-hydroxycamptothecin (HCPT) (2.5 μg/mL) for 48 hours. Expression of survivin, X-linked inhibitor of apoptosis protein (XIAP), c-inhibitor of apoptosis (IAP)-1 and c-IAP-2 were determined by western bot. (**B**) SW1116 cells were treated with HCPT for 48 hours. Expression of survivin splices was determined by real-time PCR; ^*^P <0.05, ^#^*P* >0.05, compared to the control group. **(C-D)** Knockdown of survivin and XIAP by siRNA. SW1116 cells were transfected with Survivin siRNA (**C**), and XIAP siRNA (**D**). Twenty-four hours after transfection, cells were treated with HCPT for another 48 hours. The mRNA expression of survivin (**C**) and XIAP (**D**) was measured by real-time PCR. **(E-F)** Knockdown of survivin and XIAP enhanced HCPT-induced apoptosis. SW1116 cells were transfected with Survivin siRNA (**E**), and XIAP siRNA (**F**). Then, cells were treated with HCPT for another 48 hours. Apoptosis was determined by terminal deoxynucleotidyl transferase-mediated dUTP-biotin nick end labeling (TUNEL) staining; ^*^P <0.01, compared to the control siRNA groups; ^*^P <0.01, compared to the PBS group.

[7,8]. Thus, we determined whether the activation of caspase activity and the induction of apoptosis by HCPT are due to the inhibition of IAPs. We determined the expressions of XIAP and survivin in HCPT-treated SW1116 cells. Western blot showed that HCPT significantly inhibited the expression of XIAP and survivin, but did not affect expression of c-IAP-1 and c-IAP-2 (Figure 4A), suggesting that survivin and XIAP are the targets of HCPT. In addition, we also determine the effects of HCPT on the expression of survivin splices. Real-time PCR showed that HCPT downreguated the expression of survivin 3B and survivin-ΔEx3, and upregulated the expression of survivin 2B (Figure 4B). These results suggest that HCPT has different effects on survivin splices.

To further characterize the roles of XIAP and survivin in HCPT-induced apoptosis, we silenced XIAP and survivin expression in SW1116 cells. The expressions of both proteins were downregulated by 72% 48 hours after siRNA transfections (Figure 4C and 4D). Under the same transfections, we treated SW1116 cells with HCPT and determined apoptosis. The knockdown of XIAP or survivin expression significantly increased HCPT-induced apoptosis in SW1116 cells (Figure 4E and 4F). These results suggest that the downregulation of XIAP and survivin are the major mechanisms by which HCPT induces apoptosis.

### The combination of HCPT and 5-FU synergistically induces apoptosis in colon cancer cells

**Figure 5:**
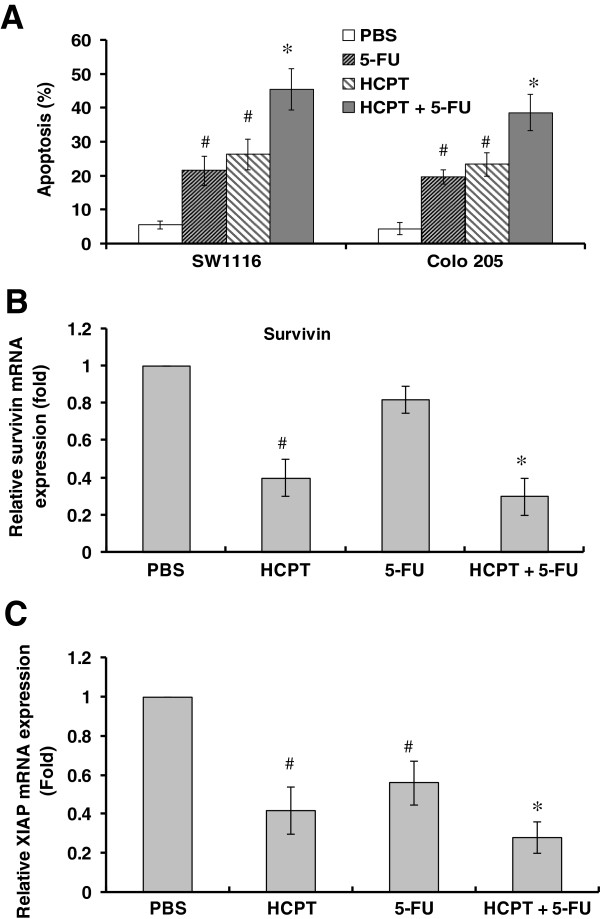
**HCPT enhances colon cancer cells to 5-FU-induced apoptosis.** (**A)**. The combination of 10-hydroxycamptothecin (HCPT) and 5-fluorouracil (5-FU) synergistically induces apoptosis. SW1116 cells and Colo 205 cells were treated with HCPT (2.5 μg/mL) or 5-FU (5 μg/mL) alone or in their combination for 48 hours. Apoptosis was determined by terminal deoxynucleotidyl transferase-mediated dUTP-biotin nick end labeling (TUNEL) staining. **(B-C)** The combination of HCPT and 5-FU synergistically downregulates expression of X-linked inhibitor of apoptosis protein (XIAP) and survivin. SW1116 cells were treated with HCPT or 5-FU alone or in their combination for 48 hours. The mRNA expression of survivin (**B**) and XIAP (**C**) were determined by real-time PCR. The data are the mean ± SD of three independent experiments. ^*^P <0.05, compared to 5-FU or HCPT alone; ^#^*P <*0.01, compared to PBS.

Clinical studies have shown that the combination of HCPT and 5-FU increases the response of patients with advanced colon cancer [22]. However, the underlying mechanisms are not fully understood. We determined the effects of the combination of HCPT and 5-FU on apoptosis and the expression of XIAP and survivin. The SW1116 cells and Colo 205 cells were treated with HCPT or 5-FU alone or in combination, and apoptosis was determined. The results showed that the combination of HCPT and 5-FU significantly increased apoptosis of SW1116 and Colo 205 cells more than HCPT or 5-FU treatment (Figure 5A). The expression of XIAP and survivin also markedly decreased in the cells treated with the combination of HCPT and 5-FU than in those treated with HCPT or 5-FU treatment alone (Figure 5B and 5C). The data indicate that the combination of HCPT and 5-FU synergistically induces apoptosis in colon cancer cells through the downregulation of XIAP and survivin.

### The combination of HCPT and 5-FU synergistically inhibits xenograft growth in colon cancer cells

**Figure 6:**
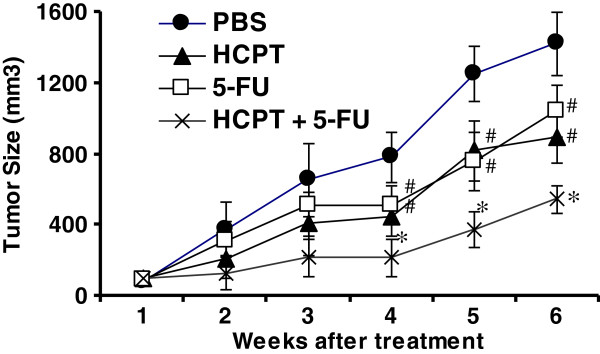
**The combination of HCPT and 5-FU synergistically inhibits tumor growth *in vivo*.** SW1116 cells were subcutaneously injected into the flank of athymic nude mice. The tumor at 50 mm^3^ to 60 mm^3^ volume was treated with 10-hydroxycamptothecin (HPCT) by intraperitoneal injection (5 mg/kg), or 5-FU (10 mg/kg) or a combination of both for 5 days. Tumor growth was measured every week after the treatment for a duration of 6 weeks. Data are the means ± SD of tumor size per mouse (n = 6). ^*^P <0.01, compared to the 5-FU or HCPT treatment alone; ^#^*P* <0.05, compared to PBS treatment.

Finally, we further investigated the effects of HCPT alone or in combination with 5-FU on tumor growth *in vivo*. SW1116 cells were injected into BABL/c nude mice. When tumor size reached 50 mm^3^ to 60 mm^3^, the mice were injected i.p. with HCPT (5 mg/kg body weight), 5-FU (10 mg/kg), the combination of HCPT and 5-FU, or PBS (control) for five days. The results showed that HCPT and 5-FU treatment alone inhibited tumor growth compared to

 the control treatment. However, the combination treatment synergistically inhibited tumor growth compared to HCPT and 5-FU treatment alone (Figure 6). The combination of HCPT and 5-FU synergistically inhibits colon cancer growth *in vivo*.

## Discussion

HCPT is an agent with an unique spectrum of anti-tumor activity mediated by a selective inhibition of eukaryotic DNA Topo-I [7,11]. This inhibition is generally thought to be mediated by the stabilization of the CPT-Topo-I-DNA cleavable complex [11]. In this study, we found that HCPT could inhibit cell proliferation and also induce apoptosis in colon cancer cells. HCPT induced apoptosis through the intrinsic and extrinsic apoptotic pathways and the downregulation of survivin and XIAP expression. HCPT and 5-FU synergistically induced apoptosis, downregulated the expressions of

 survivin and XIAP and inhibited tumor growth *in vivo*. Our results demonstrate that the important apoptotic mechanisms are related to the anticancer effects of HCPT independent from the inhibition of Topo-I.

Two main pathways of apoptosis have been characterized to date: one is triggered by death receptors (extrinsic pathway) [23,24], and the other is the mitochondrial pathway (intrinsic pathway), which is the main pathway used for stress-induced apoptosis [25,26]. In the first pathway, the utilization of adaptors in engaging receptors leads to the activation of apical caspases, such as caspase-8 [23,24]; in the second pathway, mitochondria have a central role

 in initiating apoptosis [27]. Studies have shown that camptothecin and its analogs can induce apoptosis in human leukemic cells, colon, prostate, and breast cancer cells as well as glioma cells [11]. Irinotecan, a Topo-I inhibitor, induces apoptosis of liver cancer cells through the downregulation of mutant p53 and the upregulation of Bax [28]. Irinotecan also may increase p53 transcriptional activity and induce p53 expression in wild-type p53-expressing HCT-116 colon cancer cells [29]. A previous study showed that low-dose HCPT induced apoptosis through the activation of caspase 3 [15]. Our results showed that HCPT treatment increased the activities of caspase 3. Western blot further showed that HCPT could induce the cleavage of caspase 3, 7, 8, 9. Our results demonstrate that HCPT induces apoptosis of colon cancer cells through both intrinsic (mitochondrial) and extrinsic (non-mitochondrial) pathways.

IAPs are endogenous inhibitors of caspases and are associated with chemo-resistance in some cancer cells, including colon cancer [30]. Survivin and XIAP directly inhibit the activities of caspase 3. Furthermore, the targeted inhibition of XIAP or survivin genes has been shown to directly sensitize cancer cells to apoptosis induced by various conventional chemotherapeutic drugs [31,32]. In this study, we found that HCPT could downregulate expressions of XIAP and survivin, resulting in the activation of caspase 3 enzyme activity and the execution of apoptosis. Moreover, the downregulation of XIAP or survivin enhanced the apoptosis induced by HCPT. The results document that HCPT-induced degradation of XIAP and survivin is the key event that contributes to HCPT-induced apoptosis. In addition, we also found that HCPT has different effects on the expression of surviving splices. It has reported that the expression of survivn-ΔEx3 and survivin-3B were associated with lymphoid metastasis and Dukes grade [33] and that survivin 2B has a function against survivin anti-apoptosis [34]. Our results showed that HCPT downregulated expression of survivin-ΔEx3 and survivin-3B and upregulated expression of survivin-2B. Our results are similar to the previous report that doxorubicin upregulated expression of survivin-2B and downregulated expression of survivin-ΔEx3 [34], suggesting that survivin is a major target for HCPT-anti-tumor effects.

Topo-I inhibitor has been used to treat advanced colon cancer when combined with 5-FU [35]. However, the effect of the combination of HCPT and 5-FU on colon cancer growth remains unclear. Our results showed that the combination of HCPT and 5-FU synergistically induced apoptosis, downregulated the expression of XIAP and survivin and inhibited colon cancer growth. Our study elucidates a new mechanism by which the combination of HCPT and 5-FU treats colon cancer.

In summary, our current study demonstrates that HCPT induced cell apoptosis and inhibited cell growth in human colonic cancer cells. The HCPT-induced cell apoptosis and the inhibition of growth may involve the activation of intrinsic and extrinsic apoptotic pathways and the downregulation of expression of XIAP and survivin. The combination of HCPT and 5-FU synergistically induces apoptosis, downregulates XIAP and survivin expression and inhibits xenograft tumor growth. Our findings in the present study offer a significant groundwork for future essential clinical application.

## Conclusions

Our results elucidate new mechanisms of HCPT antitumor by the downregulation of survivin and XIAP expression. The combination of HCPT with 5-FU or IAP inhibitors may be a potential strategy for colon cancer treatment.

## Abbreviations

ANOVA: Analysis of variance; CRC: Colorectal cancer; CPC: Camptothecin; CT: Cycle threshold; DMSO: Dimethyl sulfoxide; dUTP: 2´-deoxyuridine 5´-triphosphate; FBS: Fetal bovine serum; 5-FU: 5-fluorouracil; HCPT: 10-hydroxycamptothecin; IAP: Inhibitor of apoptosis; i.p: Intraperitoneal; MTT: 3(4,5 dimethylthiazol)-2,5 diphenyltetra-zolium; PARP: Poly (ADP-ribose) polymerase; PBS: Phosphate-buffered saline; PCR: Polymerase chain reaction; RPMI: Roswell Park Memorial Institute; s.c: Subcutaneously; Topo: Topoisomerase; TUNEL: Terminal deoxynucleotidyl transferase-mediated dUTP-biotin nick end labeling; XIAP: X-linked inhibitor of apoptosis protein

## Competing interests

The authors declare that they have no competing interests.

## Authors’ contributions

BJF and YW designed the study, performed the experiments and wrote the manuscript. ALC contributed to analyzing the data, discussing the results and editing the manuscript. All authors read and approved the final manuscript.
